# Acute and long-term procedural outcomes, arrhythmia recurrence and mortality after supraventricular arrhythmia ablation: a comparative study of fluoroscopy and zero-fluoroscopy guided techniques

**DOI:** 10.3389/fcvm.2025.1582753

**Published:** 2025-07-10

**Authors:** Kamilla Luca Dávid, Balázs Polgár, Péter Bógyi, Zsolt Bári, István Marczell, Manuella Bogdan, Zalán Gulyás, Mirjam Franciska Turáni, Judit Papp, Előd János Zsigmond, Emese Tóth-Zsámboki, Gábor Zoltán Duray

**Affiliations:** ^1^ Department of Cardiology, Central Hospital of Northern Pest—Military Hospital, Budapest, Hungary; ^2^ Doctoral School of Clinical Medicine, Cardiovascular Medicine and Research Division, Semmelweis University, Budapest, Hungary; ^3^ Department of Cardiology, Gottsegen National Cardiovascular Center, Budapest, Hungary; ^4^ Heart and Vascular Center, Semmelweis University, Budapest, Hungary

**Keywords:** zero-fluoroscopy catheter ablation, supraventricular tachycardia, threedimensional electroanatomic mapping system, learning-curve, long-term mortality, recurrence rate

## Abstract

**Background and aims:**

Zero-fluoroscopy (ZF) catheter ablation eliminates radiation exposure via use of 3-dimensional electroanatomical mapping. We aimed to assess safety and efficacy of ZF catheter ablation in the treatment of supraventricular tachycardias (SVTs), examine learning-curve characteristics and to evaluate long-term results and mortality.

**Methods:**

We analysed clinical characteristics, procedural and follow-up data of 605 consecutive patients undergoing catheter ablation for SVT (atrioventricular nodal re-entry tachycardia, *n* = 297; atrial flutter, *n* = 241 and accessory pathway mediated tachycardia, *n* = 67) between June 2017 and September 2021. Procedures were either guided by conventional fluoroscopy (F, *n* = 223) or by EnSite Precision mapping system (ZF, *n* = 382) based on decision of the operating physician.

**Results:**

Acute procedural success rate exceeded 98% across all arrhythmia groups for both ZF and F techniques (ZF: 99%, F: 100%, *p* = NS). 63% of patients underwent ZF procedures. Complication rate was low (0.66%), occurring only in the F group. Conversion rate to fluoroscopy was 7.8%. ZF procedures took an average of 5.1 min longer (ZF: 64.5 ± 24.3 min vs. F: 59.4 ± 29 min, *p* < 0.05), however ZF procedure times were reduced over time. At 3.2 years, total mortality was 7% with no significant difference between ZF and F. Deaths were not related to the procedures. Atrial flutter showed significantly higher recurrence in ZF compared to F (83% vs. 94%, *p* < 0.005).

**Conclusion:**

Catheter ablation of SVTs using zero-fluoroscopy approach have similar acute success, complication and mortality rate as conventional fluoroscopic interventions. However, we detected significantly higher long-term arrhythmia recurrence after ZF ablation of atrial flutter, meriting further investigation.

## Introduction

Cardiac catheter ablation has emerged as a crucial therapeutic intervention in the field of cardiac electrophysiology for the management of various arrhythmias and being considered as the gold standard therapy for symptomatic and recurrent supraventricular tachycardia (SVT) (1, 2). Traditionally, electrophysiology procedures were performed under the guidance of fluoroscopy. It has been an integral component of the procedure, providing real-time imaging guidance during catheter manipulation and lesion creation. However, the use of x-ray exposes both patients and healthcare providers to ionizing radiation, which carries inherent risks since no magnitude of radiation is free from harmful biological effect (3, 4). The American College of Cardiology (ACC) introduced the core principle of “as low as reasonably achievable” (ALARA) which propagate the minimization of non-negligible radiation-induced injury hazard in every interventional lab (5). Personnel protective shielding is in use to attenuate scatter x-ray, but it might lead to increased prevalence of cervical and lumbar spondylosis among electrophysiologists (6).

In recent years, a paradigm shift has occurred with the introduction of zero fluoroscopy catheter ablation techniques since three-dimensional (3D) electroanatomical mapping (EAM) systems, contact force sensing catheters and intracardiac echocardiography (ICE) have become widely available. By leveraging alternative imaging modalities and advanced mapping technologies, zero fluoroscopy techniques offer the potential to eliminate radiation exposure and improve patient care in the cardiac electrophysiology lab (7).

Several studies have appraised the use of near-zero or zero-fluoroscopic approach with excellent success and low complication rates (8–11). Nevertheless, current data and guidelines do not provide precise recommendation to support the decision of the operator regarding the use of fluoroscopy. Currently, just a few of the studies enrolled significant number of patients with zero-fluoroscopic approach (ZF) catheter ablation. Furthermore, the patient follow-up is often not longer than 6 or 12 months, thus it is challenging to assess the recurrence rate and long-term mortality of different cardiac arrhythmias. The clinical implementation of zero fluoroscopy catheter ablation presents challenges. Healthcare providers must overcome the learning curve associated with new imaging and navigation technologies. Additionally, certain complex cases may still require supplementary imaging, and alternative approaches may be necessary in specific situations. Since these difficulties are not technical, rather experiential, it would be beneficial to investigate the operator's learning curve of using zero-fluoroscopy technique.

To our knowledge, this is the first large-scale, single-center study from Central-Eastern Europe to report long-term outcomes of zero-fluoroscopy catheter ablation in supraventricular tachycardias. Our analysis includes over 600 patients with a mean follow-up of more than three years and provides arrhythmia-specific recurrence data for AVNRT, typical atrial flutter and accessory pathway-mediated tachycardia. In addition, we offer a structured assessment of the operator learning curve during ZF implementation, which has rarely been reported with such detail in previous literature.

Therefore, the main aim of our study was to assess the acute and long-term safety, efficacy, feasibility and mortality of near-zero and zero-fluoroscopy approach for catheter ablation of SVTs using the EnSite Precision™ mapping system compared to conventional fluoroscopy approach in an extended population. We also analysed the dynamics of zero-fluoroscopy strategy learning curve at our tertiary center.

## Methods

### Patient enrollment

We have retrospectively analysed a cohort of 605 consecutive patients, admitted at our department between 2017 June and 2021 September for catheter ablation of atrioventricular nodal re-entry tachycardia (AVNRT), atrial flutter (AFL) or accessory pathway induced tachycardia (AP). At our center, ZF strategy was adopted at the start of the study period. The AP group included—atrioventricular re-entrant tachycardia (AVRT), Wolff-Parkinson-White (WPW)-syndrome and other AP related tachycardia. Patients with catheter ablation for atrial fibrillation (AF) were excluded. Presence of cardiac implantable electronic device (CIED), chronic heart-, lung- or kidney disease were not exclusion criteria.

Data on patient characteristics and procedural details were collected from the hospital's data collecting system. All patients provided written informed consent before undergoing catheter ablation. Our research protocol was approved by the Institutional Ethics Committee and followed the principles of Declaration of Helsinki. All patients underwent a pre-procedural clinical examination, routine laboratory analysis and electrocardiography (ECG).

### Intervention strategies

In all patients, an electrophysiological study was performed and followed by catheter ablation of the target. Physicians were free to choose their preferred method: fluoroscopy-guided or zero-fluoroscopy procedure depending on technical background, personal preference, patient and arrhythmia characteristics. Patients with AVNRT, AFL and AP were subsequently categorized into distinct groups. Conventional, fluoroscopy-guided ablation comprised the fluoroscopy (F) group. Patients with initial decision to perform the procedure fully without fluoroscopic guidance comprised the zero-fluoroscopic (ZF) group. During the intervention physicians were allowed to convert the procedure from zero fluoroscopy procedure to fluoroscopy-assisted procedure in order to keep the highest safety and efficacy profile of the procedures. These patients comprised the Conversion (C) group.

### Catheter positioning and ablation

Procedures were performed in local anesthesia via femoral vein access in all patients. In selected cases different level of conscious sedation was applied by using bolus administration of fentanyl and midazolam. Electrophysiological studies were performed using standard pacing protocols. The placement of the catheters was performed under fluoroscopy in the F group. In patients with zero fluoroscopy approach, a 3D EAM system (EnSite NavX, EnSite Velocity, EnSite Precision, Abbott, St. Paul, MN, USA) was used. The number, type and position of the catheters were freely selected by the physician matching the actual arrhythmia target. Strategies regarding different methods such as point-by-point or dragging ablation for isthmus ablation were freely selected by the physicians. Typically, isthmus ablation was performed by an 8 mm gold tip ablation catheter, with pull-and burn method with at least 30 s RF energy delivery at a specific position. Advanced lesion guided methods such as ablation index or lesion size index were routinely not used as contact force catheter was rarely used for isthmus ablation or slow pathway ablation. No additional catheter was used only to aid electroanatomical mapping. ICE or CT fusion were not routinely used during the procedures.

### Procedural endpoints

Endpoints for successful procedure were based on generally accepted criteria such as non-inducibility and lack of slow pathway conduction or maximal one AV nodal echo beat for AVNRT, while elimination of the conduction in the cavotricuspidal area for AFL. In case of AP dependent tachycardia, the elimination of AV and VA conduction across the accessory pathway was accepted as procedural endpoint.

### Procedural complications

Major complications were events directly associated with the catheter ablation procedure, necessitating intervention, extended hospital stay, and/or exerting an adverse impact on the patient's long-term health. Minor complications involved transient low- or high-degree atrioventricular block, which resolved during or after the procedure, pericardial effusion without hemodynamic compromise necessitating no intervention, and other adverse events directly associated with the catheter ablation procedure that did not meet the criteria for major complications.

### Follow-up

After the procedure, all patients were provided with post-procedural instructions delineating further actions in case of recurrence. Subsequently, during 3-months scheduled outpatient visits, patients underwent comprehensive clinical examinations and had a 12-lead ECG recorded. 12-months-, last medical follow-up data and mortality data were gathered from the National eHealth Infrastructure (EESZT) system. Approximately one-third of the population was followed in the outpatient clinic of the operating center and two-third in other institutions. Postoperative atrial fibrillation was identified based on clinical follow-up medical documentation, systematic screening was not performed. It is important to note that in certain cases, we did not receive follow-up data. Results such as success rate or recurrence rate were calculated based on all available information.

### Statistical analysis

Continuous data were reported as mean with standard deviation or median with interquartile ranges (IQR) when distributions were not normal. Categorical variables were presented as frequencies and percentages. Between group differences were compared with two sample t-test or Mann–Whitney *U*-test for continuous variables, while we used *χ*^2^-test os Fisher's exact test for categorical variables. All tests were two sided, and a *p*-value of <0.05 was considered statistically significant. We used Kaplan–Meier survival analysis and log-rank test for cumulative survival data and for arrhythmia-free survival data. The statistical analyses were performed with the SPSS 28.0.1.0 (IBM, Armonk, NY, USA) software and TIBCO Statistica software.

## Results

### Patient demographics

Within the enrolled 605 patients, 241 cavotricuspidal isthmus (CTI) ablation of typical AFL, 297 slow-pathway ablations due to AVNRT and 67 AP ablation were performed.

Majority of the patients underwent an electrophysiological procedure by using ZF strategy (in total 382, 63%). Detailed baseline clinical characteristics are presented in Table 1.

**Table 1:** Clinical data and demographic parameters of the complete patient population and comparison of baseline characteristics between the three arrhythmia types (atrial flutter, atrioventricular nodal re-entry tachycardia, accessory pathway induced tachycardia) and between the F and ZF groups.

Demographic data	Atrial flutter (*n* = 241)			Atrioventricular nodal re-entry tachycardia (*n* = 297)			Accessory pathway (*n* = 67)		
	Group ZF + C	Group F	*p*	Group ZF + C	Group F	*p*	Group ZF + C	Group F	*p*
	(*n* = 118)	(*n* = 123)		(*n* = 214)	(*n* = 83)		(*n* = 50)	(*n* = 17)	
Male/female, *n* (%)	92/26 (78/22)	94/29 (76/24)	0.837	89/125 (42/58)	35/48 (42/58)	0.939	36/14 (72/28)	11/6 (65/35)	0.579
Age, years	65.8 ± 12	67.2 ± 13.3	0.189	55.4 ± 17.8	63.2 ± 16	**0**.**015**	43.0 ± 19.5	44.394 ± 17.7	0.655
BMI, (kg/m^2^)	29.4 (26.2–34.1)	27.8 (25.2–31.2)	**0**.**029**	26.5 (23.4–30.1)	26.5 (23.6–31.2)	0.518	24.6 (22.1–29.2)	28.3 (25.5–33.4)	**0** **.** **025**
Hypertension, *n* (%)	100 (85)	99 (80)	0.384	88 (41)	51 (61)	**0**.**002**	16 (32)	10 (59)	**0** **.** **050**
Diabetes mellitus, *n* (%)	41 (35)	28 (23)	**0**.**040**	29 (14)	21 (25)	**0**.**015**	2 (4)	0 (0)	0.403
Stroke, *n* (%)	8 (7)	6 (5)	0.528	3 (1)	1 (1)	0.895	0 (0)	0 (0)	–
CIED, *n* (%)	10 (8.5)	18 (15)	0.136	3 (1)	6 (7)	**0**.**009**	0 (0)	0 (0)	–
Valvular heart disease, *n* (%)	7 (6)	10 (8)	0.505	3 (1)	2 (2)	0.5447	1 (2)	0 (0)	0.557
Ischaemic heart disease, *n* (%)	36 (30.5)	39 (32)	0.841	17 (8)	7 (8)	0.890	5 (10)	1 (6)	0.608
Chronic heart failure, *n* (%)	31 (26)	25 (20)	0.275	4 (2)	4 (5)	0.159	1 (2)	0 (0)	0.557
Pulmonary disorder, *n* (%)	18 (15)	14 (11)	0.376	14 (6.5)	9 (11)	0.213	4 (8)	0 (0)	0.229
GERD, *n* (%)	11 (9)	11 (9)	0.919	18 (8)	9 (11)	0.513	4 (8)	3 (18)	0.261
OSAS, *n* (%)	6 (5)	3 (2)	0.279	2 (1)	1 (1)	0.835	2 (4)	0 (0)	0.403
Hypothyreosis, *n* (%)	13 (11)	9 (7)	0.319	15 (7)	5 (6)	0.761	1 (2)	1 (6)	0.417
Hyperthyreosis, *n* (%)	5 (4)	4 (3)	0.687	3 (1)	2 (2)	0.545	0 (0)	0 (0)	–
Dyslipidaemia, *n* (%)	16 (14)	17 (14)	0.953	22 (10)	10 (12)	0.659	4 (8)	2 (12)	0.639
Anxiety disorder, *n* (%)	4 (3)	3 (2)	0.660	12 (6)	4 (5)	0.787	3 (6)	1 (6)	0.986
Renal failure, *n* (%)	8 (7)	8 (6.5)	0.932	4 (2)	2 (2)	0.766	1 (2)	0 (0)	0.557
Ejection fraction, (%)	53 (40–61)	53.5 (45–60)	0.547	63 (60–66)	61 (58–68)	0.696	60 (60–65)	65 (62.5–67.5)	0.059
Electrocardiographic parameters									
Pre-ECG									
Sinus rhythm	57 (48)	46 (37)	0.087	213 (99.5)	79 (95)	**0** **.** **009**	46 (92)	15 (88)	0.639
Atrial flutter	54 (46)	73 (59)	**0** **.** **035**	0 (0)	0 (0)	–	1 (2)	1 (6)	0.417
Atrial fibrillation	5 (4)	2 (2)	0.228	0 (0)	0 (0)	–	1 (2)	0 (0)	0.557
Pacemaker rhythm	0 (0)	2 (2)	0.164	0 (0)	1 (1)	0.108	0 (0)	0 (0)	–
Other*	1 (1)	0 (0)	0.306	1 (0.5)	3 (4)	**0** **.** **035**	2 (4)	0 (0)	0.403
Bundle branch block									
RBBB	18 (15)	22 (18)	0.583	7 (3)	4 (5)	0.526	3 (6)	1 (6)	0.986
LBBB	10 (8)	12 (10)	0.730	3 (1)	1 (1)	0.895	2 (4)	0 (0)	0.403
LAFB	5 (4)	4 (3)	0.687	5 (2)	4 (5)	0.263	0 (0)	0 (0)	–
PQ (msec)	160 (140–180)	160 (140–180)	0.912	160 (120–160)	160 (140–160)	0.060	120 (100–140)	140 (110–160)	0.535
QRS (msec)	92 (80–120)	97.5 (80–120)	0.891	80 (80–90)	80 (80–100)	0.236	95 (80–120)	95 (80–120)	0.889
Medical therapy									
Antiplatelet and anticoagulant therapy									
ASA, 100 mg									
at admission, (%)	12 (10)	18 (15)	0.294	39 (18)	24 (29)	**0**.**043**	3 (6)	3 (18)	0.146
at discharge, (%)	7 (6)	17 (14)	**0** **.** **041**	199 (93)	75 (90)	0.447	41 (82)	14 (82)	0.974
Clopidogrel, 75 mg/150 mg									
at admission, (%)	9 (8)	5 (4)	0.237	12 (6)	5 (6)	0.890	2 (4)	1 (6)	0.746
at discharge, (%)	8 (7)	5 (4)	0.351	13 (6)	3 (4)	0.399	2 (4)	1 (6)	0.746
VKA									
at admission, (%)	15 (13)	23 (19)	0.202	4 (2)	1 (1)	0.689	0 (0)	0 (0)	–
at discharge, (%)	14 (12)	25 (20)	0.075	5 (2)	1 (1)	0.534	1 (2)	2 (12)	0.093
NOAC									
at admission, (%)	79 (67)	74 (60)	0.274	7 (3)	5 (6)	0.280	4 (8)	0 (0)	0.229
at discharge, (%)	97 (82)	87 (71)	**0** **.** **036**	8 (4)	3 (4)	0.960	4 (8)	0 (0)	0.229
LMWH									
at admission, (%)	6 (5)	5 (4)	0.705	3 (1)	3 (4)	0.224	0 (0)	0 (0)	–
at discharge, (%)	1 (1)	2 (2)	0.586	0 (0)	1 (1)	0.108	0 (0)	0 (0)	–
Antiarrhythmic therapy									
Beta-blocker									
at admission, (%)	103 (87)	103 (84)	0.435	122 (57)	56 (67.5)	0.099	19 (38)	10 (59)	0.134
at discharge, (%)	105 (89)	108 (88)	0.775	82 (38)	44 (53)	**0** **.** **022**	15 (30)	4 (23.5)	0.609
Propafenone									
at admission, (%)	13 (11)	17 (14)	0.510	18 (8)	8 (10)	0.737	3 (6)	4 (24)	0.092
at discharge, (%)	7 (6)	15 (12)	0.092	3 (1)	1 (1)	0.895	3 (6)	1 (6)	0.986
Amiodarone									
at admission, (%)	26 (22)	17 (14)	0.096	2 (1)	2 (2)	0.322	1 (2)	0 (0)	0.557
at discharge, (%)	26 (22)	21 (17)	0.415	3 (1)	2 (2)	0.545	2 (4)	0 (0)	0.403
Calcium Channel Blockers (Nondihydropyridine)									
at admission, (%)	0 (0)	1 (1)	0.326	8 (4)	1 (1)	0.253	1 (2)	0 (0)	0.557
at discharge, (%)	0 (0)	0 (0)	–	1 (0.5)	0 (0)	0.533	1 (2)	0 (0)	0.557

We collected demographic data about patients underwent RF ablation. We also added information about the antiarrhythmic therapy of patients to see the changes after ablation procedures. ZF and C patients are shown together.

Significant *p*-values are indicated in bold.

BMI, body mass index; CIED, cardiac implantable electronic devices; GERD, gastroesophageal reflux disease; OSAS, obstructive sleep apnoea syndrome; RBBB, right bundle branch block; LBBB, left bundle branch block; LAFB, left anterior fascicular block; ASA, acetylsalicylic acid; VKA, vitamin K antagonist; NOAC, non-vitamin K antagonist oral anticoagulants; LMWH, low molecular weight heparin.

* Other rhythms classified as AVNRT, AVRT, WPW syndrome, others.

The AFL group exhibited a male predomination, whereas females were predominant in the AVNRT group, aligning with established international distributions for these specific arrhythmias (12). Subgroup analysis revealed significantly younger patients in the ZF + C subgroup compared to the F subgroup within the AVNRT group (55.4 ± 17.8 years vs. 63.2 ± 16 years, *p* < 0.05), a phenomenon not observed in the AFL or AP groups.

Comorbidities were more prevalent in the AFL group, with occurrence of chronic heart failure in 23% and ischemic heart disease in 31%. Importantly, there were no significant differences in incidence of major comorbidities between the ZF + C and F subgroups, suggesting that comorbidities have not influenced the operator's decision of which approach to choose. Data are provided in Table 1.

Most ablation procedures occurred in sinus rhythm (456 patients in total, 75%); while in the AFL group atrial flutter rhythm was present in 53% of cases. The subgroup analysis demonstrated that in the presence of ongoing arrhythmia patients were more likely to undergo fluoroscopic ablation (AFL: 59%, vs. 46% *p* = 0.035). Cardiac implantable electronic devices were more often present in AVNRT (6% vs. 3%, *p* = 0.009) and AFL patients (18% vs. 10%, *p* = 0.136) targeted with fluoroscopy method.

### Procedural data

The acute procedural success rate of both techniques was equally high, exceeding 98% across all arrhythmia groups (AFL: 99%; AVNRT: 100%; AP: 98.5%). Totally zero-fluoroscopy procedures (not including conversion cases) reached 99% acute success rate, while all procedures were successful in the F group (*p* = NS).

Detailed procedural data are provided in Table 2. Acute minor procedural complication rate was low, 0.6% in total (AFL: 0.4%, AVNRT: 1%, AP: 0%); all complications occurred in the F group. The four complications consist of two cases of 1st-grade atrioventricular block, one case of 3rd-grade atrioventricular block, and an accidental fast-pathway disruption during slow-pathway ablation. The patient with fast-pathway disruption required a DDD-pacemaker implantation after the ablation procedure. No major complications related to the procedure were observed, including cardiac tamponade, bleeding requiring transfusion, significant puncture site issues, thromboembolism, stroke and symptomatic phrenic nerve palsy.

**Table 2:** Procedural data of RF ablations in patients with AVNRT, AFL and AP related arrhythmias. Procedures requiring conversion from ZF to F approach (C) are shown separately.

Procedural data	Atrial flutter			Atrioventricular nodal re-entry tachycardia			Accessory pathway		
	ZF	F	C	ZF	F	C	ZF	F	C
	(*n* = 102)	(*n* = 123)	(*n* = 16)	(*n* = 201)	(*n* = 83)	(*n* = 13)	(*n* = 32)	(*n* = 17)	(*n* = 18)
	42%	51%	7%	68%	28%	4%	48%	25%	27%
Acute success (%)	100 (98)	123 (100)	16 (100)	201 (100)	83 (100)	13 (100)	31 (97)	17 (100)	18 (100)
Acute complication (%)	0 (0)	1 (1)	0 (0)	0 (0)*	3 (4)	0 (0)	0 (0)	0 (0)	0 (0)
Procedure time (min)	58 (45–70)*	50 (37–66)	71.5 (47.5–86)	60.5 (50–80)*	53.5 (40–70)	80 (55–96)	70 (55–85)	75 (60–113)	85 (73.5–109)**
Fluoroscopy time (sec)	0.0 (0.0–0.0)*	155 (108–283)	145.5 (121.5–298)**	0.0 (0.0–0.0)*	148 (90–217)***	58.5 (24–85.5)**	0.0 (0.0–0.0)*	301 (153–366)***	107 (63–205)**
Hospitalization time (days)	1.0 (1.0–2.0)	2.0 (1.0–3.0)	1.0 (1.0–5.5)	1.0 (1.0–1.0)	1.0 (1.0–2.0)	1.0 (1.0–1.0)	1.0 (1.0–1.5)	1.0 (1.0–1.0)	1.0 (1.0–1.0)
Redo procedure, *n* (%)	7 (7)	8 (6.5)	0 (0)	10 (5)	3 (4)	0 (0)	1 (3)	2 (12)	1 (6)
Acute procedure, *n* (%)	21 (21)	27 (22)	4 (25)	26 (13)	6 (7)	2 (15)	3 (9)	2 (12)	0 (0)
Elective procedure, *n* (%)	81 (79)	96 (78)	12 (75)	175 (87)	77 (93)	11 (85)	29 (91)	15 (88)	18 (100)

We detected significant differences between procedure-, and fluoroscopy times. Our findings show that ZF procedures lasted longer, except the AP group. Conversional procedures were the longest in every arrhythmia group. Fluoroscopy time, as expected, decreased significantly in every ZF cases.

Normally distributed parameters are given in a mean ± standard error of the mean (SEM) format, parameters with non-normal distributions were shown as median and interquartile ranges (IQR). Categorical values are given as *n* and percentage of the corresponding group (%).

* The difference is significant between the ZF and F groups.

** The difference is significant between the ZF and C groups.

*** The difference is significant between the F and C groups.

In ZF groups longer procedural durations were recorded in comparison to F groups, particularly within AFL [58 (45–70) min vs. 50 (37–66) min, *p* < 0.05] and AVNRT [60.5 (50–80) min vs. 53.5 (40–70) min, *p* < 0.05] categories. However, it is noteworthy that the mean procedural time difference was only 5.1 min. Disparities in procedural time for each arrhythmia are described in Table 2.

As expected, fluoroscopy time demonstrated significant differences between ZF, C and F procedures across all arrhythmia subgroups.

We found no significant difference regarding hospitalization duration (Table 2).

### Procedural data in patients with conversion

Conversion rate to fluoroscopy guidance from zero-fluoroscopy strategy was 7.8%: 6% in AVNRT, 13.6% in AFL and 36% in AP (Table 2). Procedure times were the longest in these subgroups compared to ZF or F.

However, it is important to note that in case of conversion, fluoroscopy time remained almost the same [AFL: median 145.5 (121.5–298) sec vs. 155 (108–253) sec, *p* = NS] or even significantly lower (AVNRT: median 58.5 (24–85.5) sec vs. 148 (90–217) sec, *p* < 0.05 and AP: median 107 (63–205) sec vs. 301 (153–366) sec, *p* < 0.05) as with primary fluoroscopy strategy.

### Follow-up data

Follow-up data are presented in Table 3. The average follow-up was 1,183 ± 491 days (3.2 years) at study closure. One-third of the patients received long-term cardiac care at our center.

**Table 3:** Follow-up and mortality data of patients undergoing RF ablation.

Follow-up data	Atrial flutter			Atrioventricular nodal re-entry tachycardia			Accessory pathway		
	ZF	F	C	ZF	F	C	ZF	F	C
	*n* = 102	*n* = 123	*n* = 16	*n* = 201	*n* = 83	*n* = 13	*n* = 32	*n* = 17	*n* = 18
0–3 months									
Success, (%)	95	97	100	99	96	100	92	94	81
Original arrhythmia recurrance (%)	5	3	0	1	4	0	8	6	19
Death (%)	0	2	0	0.5	1	0	3	0	0
Other new arrhythmia									
Atrial fibrillation (%)	8	7	0	0.5	1	0	0	0	0
Atypical atrial flutter (%)	0	2	0	0	0	0	0	0	0
Palpitation (%)	3	7	6	9	8	0	4	6	6
PM implantation (%)	2	3	0	0	0	0	0	0	0
ECG									
Sinus rhythm (%)	88	89	94	97	92	100	96	100	100
PM rhythm (%)	8	6	6	3	6	0	0	0	0
Atrial fibrillation (%)	1	2	0	0	1	0	4	0	0
Atrial flutter (%)	3	3	0	0	0	0	0	0	0
Drug changes									
None (%)	82	89	94	91	96	84	96	81	75
NOAC quit (%)	9	5	0	3	1	8	4	19	25
Antiarrhythmic drug quit (%)	4	2	0	3	2	0	0	0	0
Both quit (%)	0	2	0	0	0	0	0	0	0
Dosage increase of any antiarrhythmic drug (%)	5	2	6	3	1	0	0	0	0
NOAC introduction again (%)	0	0	0	0	0	8	0	0	0
0–12 months									
Success (%)	88	96	100	98	96	100	90	87	81
Original arrhythmia recurrance (%)	12	4	0	2	4	0	10	13	19
Death (%)	1	4	6	1	1	0	6	0	0
Other new arrhythmia									
Atrial fibrillation (%)	10	10	19	2	2	0	0	0	0
Atypical atrial flutter (%)	0	2	0	0	0	0	0	0	0
Palpitation (%)	3	7	6	9	8	8	4	13	13
PM implantation (%)	2	3	0	0	0	0	0	0	0
Drug changes									
None (%)	96	97	87	95	99	100	86	100	75
NOAC quit (%)	0	1	7	1	0	0	10	0	25
Antiarrhythmic drug quit (%)	1	1	6	0	0	0	4	0	0
Both quit (%)	0	0	0	0	0	0	0	0	0
Dosage increase of any antiarrhythmic drug (%)	2	1	0	4	0	0	0	0	0
NOAC introduction again (%)	1	0	0	0	1	0	0	0	0
Last medical data									
Follow-up time (days) [Median]	791 (548–1,084)	1,342 (741–1,558)	1,131 (741–1,503	788 (457–1,234)	1,295 (541–1,707)	857 (736–1,337)	655 (141–1,135)	1,052 (434–1,566)	452 (214–650)
[Mean]	857 ± 47	1,183 ± 47	1,105 ± 126	859 ± 41	1,101 ± 72	1,037 ± 124	711 ± 129	979 ± 157	520 ± 95
Follow-up type									
Operating center (%)	35	40	38	32	34	22	29	74	44
General cardiology (%)	41	28	38	38	35	22	38	13	25
Other (e.g., internal or emergency medicine unit, etc.) (%)	24	32	24	30	31	56	33	13	31
Success (%)	83	94	100	97	96	100	90	87	81
Original arrhythmia recurrance (%)	17	6	0	3	4	0	10	13	19
Death (%)	11	15	19	2	3	0	12	0	0
Stroke (%)	0	1	0	0	0	0	0	0	0
Cancer appearance/controll (%)	6	3	0	1	0	0	0	0	0
Other new arrhythmia									
Atrial fibrillation (%)	14	15	25	2	2	0	0	0	0
Atypical flutter (%)	0	2	0	0	0	0	0	0	0
Palpitation (%)	4	7	13	9	8	0	4	13	6
PM implantation (%)	13	11	0	3	1	0	0	0	0
Cardioversion (%)	1	0	0	0	0	0	0	0	0
Number of survived days for mortality estimation at study closure									
Mean of time (days)	1,032 ± 47	1,259 ± 45	1,232 ± 124	1,109 ± 32	1,508 ± 53	1,144 ± 117	961 ± 76	1,383 ± 112	1,064 ± 100
Median of time (days)	957 (666–1,393)	1,412 (854–1,657)	1,219 (912–1,681)	1,121 (787–1,394)	1,638 (1,402–1,812)	1,176 (742–1,435)	957 (740–1,200)	1,495 (1,172–1,716)	1,089 (771–1,394)

We collected follow-up data at 3-, and 12-months. We used the National eHealth Infrastructure (EESZT) system and evaluated each patient's last medical consultation to collect further information (last medical data). One third of the population was followed at our Cardiology Unit. We collected mortality data, where the AFL group showed a relatively high mortality at last follow-up.

Normally distributed parameters are given in a mean ± standard error of the mean (SEM) format, parameters with non-normal distributions were shown as median and interquartile ranges (IQR). Categorical values are given as *n* and percentage of the corresponding group (%).

NOAC, non-vitamin K antagonist oral anticoagulants; PM, pacemaker.

#### Long-term success rate and recurrence of original arrhythmia

**Figure 1:**
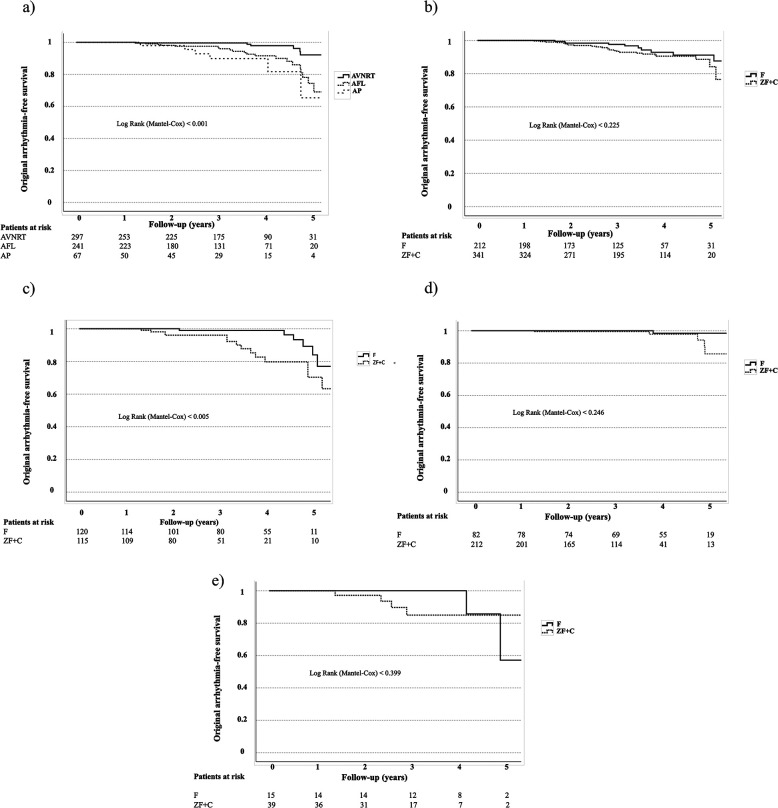
**(a)** original arrhythmia-free survival data regarding different arrhythmia types. **(b)** Original arrhythmia-free survival data regarding ZF + C/F approach during ablation in the total patient cohort. **(c)** Original arrhythmia-free survival data regarding ZF + C/F approach in the AFL group. **(d)** Original arrhythmia-free survival data regarding ZF + C/F approach in the AVNRT group. **(e)** Original arrhythmia-free survival data regarding ZF + C/F approach in the AP group.

Original arrhythmia-free survival data are provided in Figure 1a and Table 3.

The three-month procedural success rate remained high, 96.5% in average, demonstrating noteworthy efficacy across various arrhythmia types.

Within the different arrhythmia categories, early (3-months) and late (one year) original arrhythmia recurrence was the highest in the AP group (10% and 13.5%, *p* = NS, respectively), while lower recurrence rates were observed in AFL (3.8% and 7.5%, *p* = NS, respectively) and AVNRT patients (1.7% and 2.3%, *p* = NS, respectively) (Table 3). However, it is worth mentioning that the small sample size of AP cases may magnify the calculated percentage in this group relative to others. The AVNRT group displayed the most favorable, significantly superior recurrence rates at all follow-up time points, aligning with established international reports (1, 2, 13) (Figure 1a).

#### Comparison of zero-fluoroscopy and fluoroscopy approach

Original arrhythmia-free survival data for the total patient cohort are provided in Figure 1b) and Table 3.

Comparing ZF and F techniques in the total cohort, long-term success rates were similar (Figure 1b, *p* = NS).

In subgroup analysis, patients with atrial flutter showed a significantly lower long-term success rate in the ZF group compared to F group at 12-months [88% vs. 96%, *p* = 0.030 (Table 3)] and during the last follow-up (83% vs. 94%, *p* = 0.015) (Figure 1c). Most arrhythmia recurrences occurred after 3- (ZF + C) and 4-years (F) of follow up.

Long-term success rate was high in AVNRT and AP groups (Table 3). In AVNRT and AP patients, we found no difference in recurrence rates: the arrhythmia-free survival was similar between ZF and F strategies (Figures 1d,e; *p* = NS).

Concerning patients requiring conversion during operation, in case of AFL and AVNRT the long-term success rates were 100% and 100%, respectively, however the AP subgroup's long-term success rate was 81% (Table 3).

#### Clinically detected new-onset atrial fibrillation

During the 3.2 years average follow-up time, incidence of clinically detected new-onset atrial fibrillation (noAF) was 7% in the total population. The majority (86%) of noAF was detected in the AFL group (Table 3); during follow-up 15% of AFL patients developed atrial fibrillation in agreement with previously described frequent co-occurrence of these arrhythmias especially after CTI catheter ablation procedure (14, 15). Atrial fibrillation incidence was similar in ZF and F subgroups at 3-months (8% and 7%, respectively), at 12-months (10% and 10%, respectively) and at the last medical follow-up (14% and 15%, respectively).

Patients with noAF showed similar basic demographic parameters as the AFL patient group concerning age, gender distribution, BMI, incidence of hypertension, DM, ischemic heart disease and heart failure. However, concomitant pulmonary disorders were more frequent within this group [23% vs. 13% (Table 1)].

#### Cardiac implanted electronic device (CIED) during follow-up

In total 33 devices have been implanted during follow-up. Majority of procedures was performed in AFL patients [AFL: 27 cases (82%), AVNRT: 6 cases (18%)]. This indicates, that 11% (2%/year) of AFL and 2% (0.3%/year) of AVNRT patients required CIED implantation. DDD-R permanent pacemaker was used most frequently (*n* = 17). Cardiac resynchronization therapy was applied in 6 AFL cases and ICD was implanted in 5 AFL and 1 AVNRT patient. 12 implantation was executed within the first 7 days after arrhythmia ablation [DDD-R (*n* = 8); ICD (*n* = 3); AAI (*n* = 1)]; only 1 case was associated with the original procedure as an acute complication (fast-pathway damage during AVNRT ablation). The average time between ablation and device implantation was 347 ± 457 days. There was no significant difference between ZF and F procedures regarding CIED implantation. Indications were primary or secondary prevention in HFrEF patients (*n* = 12), sick sinus syndrome (*n* = 8), AV conduction disorders (*n* = 3) and tachy-brady syndrome (*n* = 10).

### Mortality and survival data

During 3-months follow-up, 1 patient in the AP, and 2–2 patients in the AFL and AVNRT groups deceased. Total mortality rate at 3-months was 0.8%. The cause of death was not related to the original arrhythmias or the procedure (left-ventricular failure; pneumonia—sepsis; acute promyelocytic leukemia; sudden cardiac death and one unknown cause) (Table 3).

At 12-months, mortality rate was 1.8% of the total population. In respect to individual mortality rates: AFL: 2.9%; AVNRT: 1%; AP: 1.5% (Table 3).

**Figure 2:**
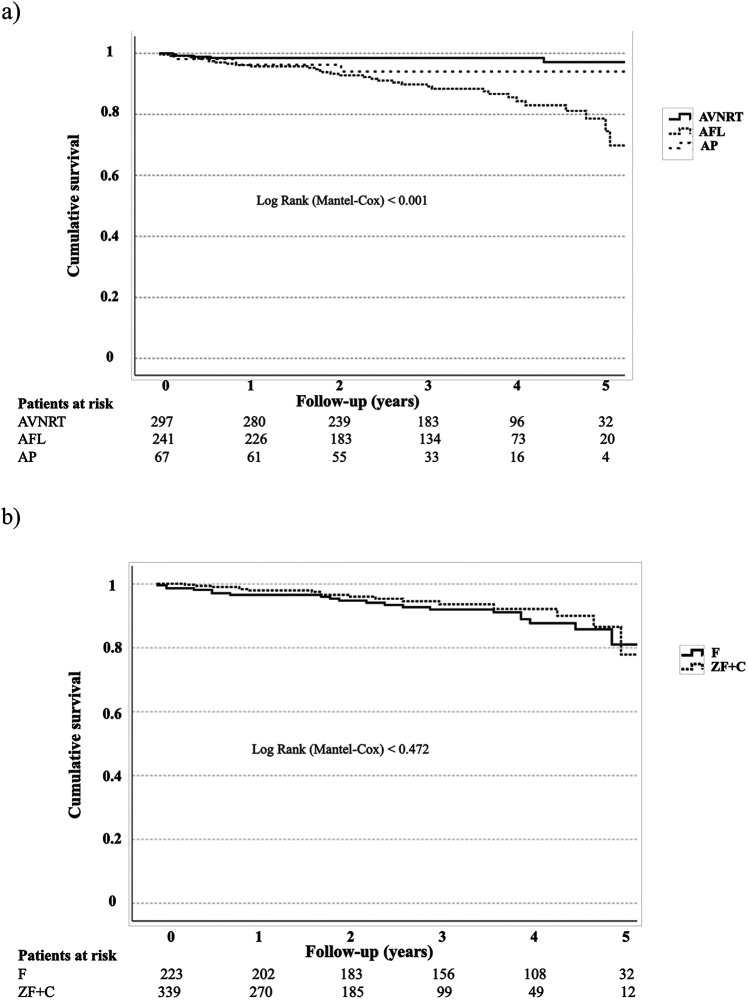
**(a)** cumulative survival data of the total population regarding different arrhythmia types. **(b)** Cumulative survival data of the total population regarding ZF + C/F approach.

The last medical data were obtained at 3.2 years follow-up in average (Table 3). There is a further increasing mortality rate: 7% of the total population died during follow-up. The increasing mortality rate can be attributed to the AFL patient cohort: majority of total death occurred in the AFL group with 13% rate of total mortality (cumulative survival data are presented in Figure 2a, *p* < 0.001). AVNRT and AP patient mortality remained low at median follow-up (2% and 6%, respectively). Cause of further deaths was septicaemia, sudden cardiac death, acute heart failure and malignant diseases. Stroke rate was low, 1%.

In respect to fluoroscopy approach, we found no significant difference concerning cumulative survival of the total patient population with F or ZF + C method (Figure 2b).

### Learning curve

#### Incidence of zero-fluoroscopy interventions

**Figure 3:**
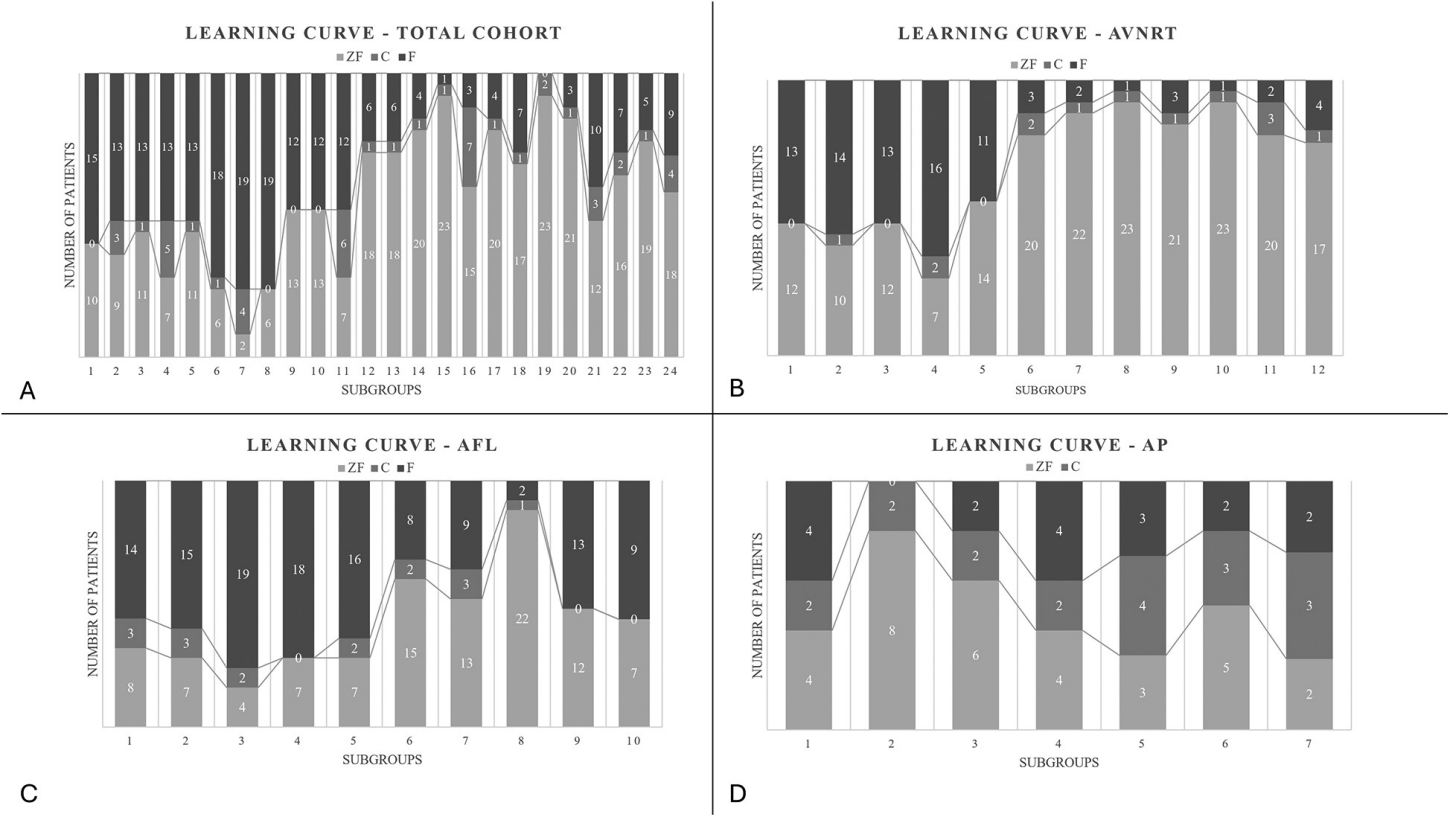
**(A)** Learning curve of the total cohort showing the increase of the zero-fluoroscopy cases with time. **(B)** Learning curve of the atrioventricular nodal re-entry tachycardia group showing increase in the frequency of ZF cases. Consecutive AVNRT patients were divided into subgroups comprising 25 individuals. Number of F/ZF and C cases are given in each subgroup. **(C)** Learning curve of the atrial flutter group showing the changes in the distribution of the techniques. Consecutive AFL patients were divided into subgroups comprising 25 individuals. Number of F/ZF and C cases are given in each subgroup. **(D)** Learning curve of the accessory pathway group showing the changes in the distribution of the techniques. Consecutive AP patients were divided into subgroups comprising 10 individuals. Number of F/ZF and C cases are given in each subgroup.

A learning-curve analysis was conducted to examine the chronological dynamics of various arrhythmia cases over the years. Initially, cases were stratified into 24 smaller consecutive cohorts, each comprised of 25 cases. As depicted in Figure 3A), there was an evident decline in number of fluoroscopy-guided procedures over time, in line with corresponding increase of zero-fluoroscopy procedures.

Frequency of interventions requiring conversion to fluoroscopy (C) varied largely in subgroups of consecutive patients (0%–28%), but its incidence did not suggest dynamics of a learning curve during our study period (Figure 3A).

Upon separating the arrhythmia cohorts for further analysis, procedural approach exhibited the most noticeable shift in the AVNRT group, which is widely regarded as one of the most straightforward arrhythmias for ZF procedure. As expected, the use of ZF significantly increased during the study period, from 50% up to 80%. The rate of conversion was the lowest within the study groups, between 0%–1% (Figure 3B).

Regarding patients with AFL, ZF approach was applied in 42% of the cases with a low conversion rate (7%). Despite the initially high fluoroscopy ratio, ZF approach was gradually adapted in this arrhythmia cohort as well (in subgroup 1–5 total ZF cases were 34%, in group 6–10 total ZF cases were 65%, Figure 3C).

The above described pattern was not observed in the accessory pathway group (Figure 3D). Fluoroscopy-guided transseptal puncture, particularly when the arrhythmia is localized to the left atrium is often required in these patients. Since ICE was not employed routinely during the study period, the practice of fluoroscopic approach was not significantly reduced over time.

#### Procedural time

**Figure 4:**
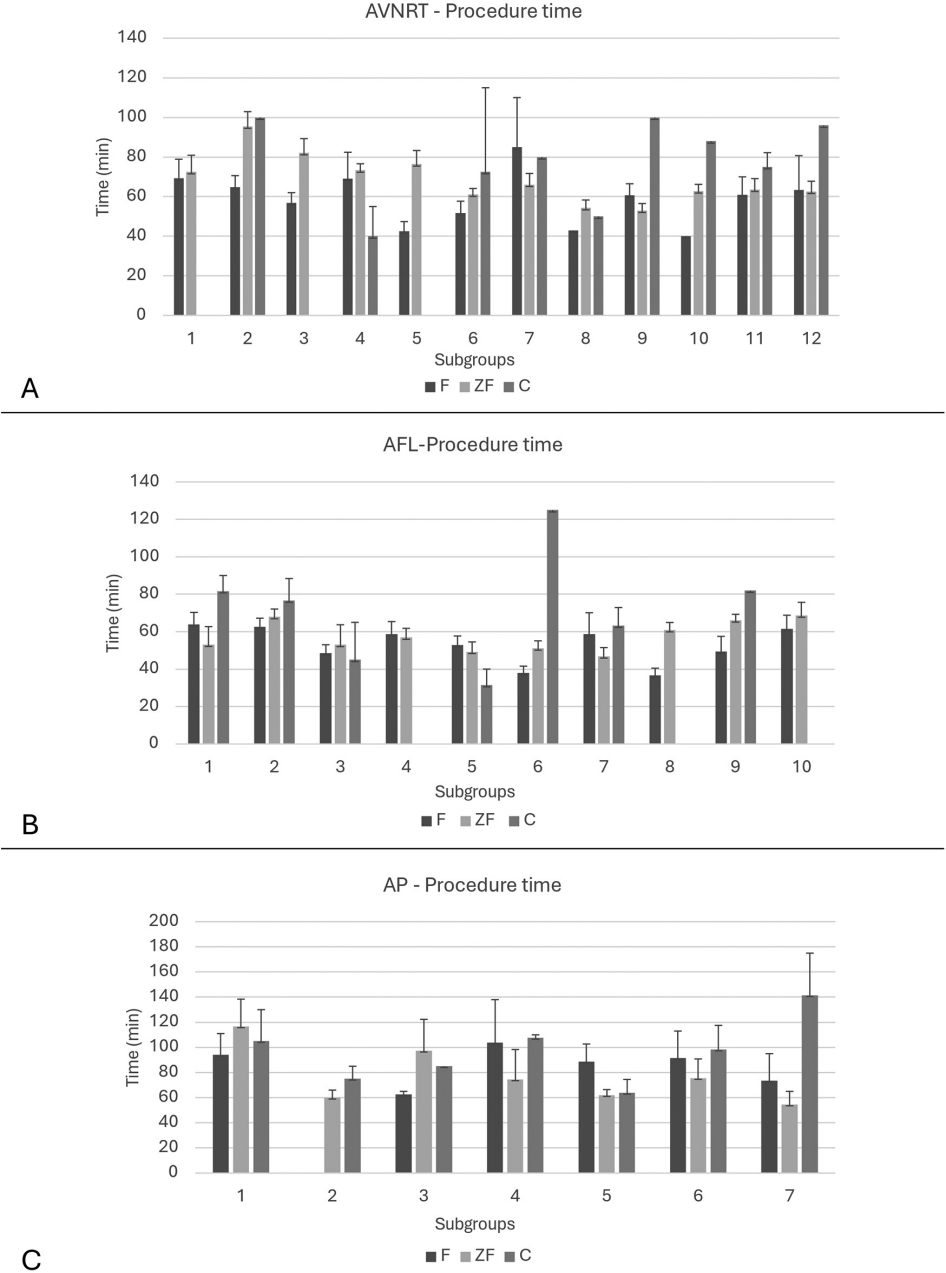
Learning curve of procedure time in **(A)** AVNRT, **(B)** AFL and **(C)** AP patient subgroups. In consecutive AVNRT and AFL patients we generated subgroups comprised of 25 patients. Consecutive AP patients were divided into 7 subgroups consisting 10 individuals. In each subgroup, average procedural times are provided for fluoroscopy-guided, zero-fluoroscopy and conversion procedures. In AVNRT and AP patients with zero-fluoroscopy approach, procedural time was reduced in the second half of our study.

In Figures 4A–C, procedural times are provided separately for each arrhythmia subgroup.

Interestingly, procedural times were significantly reduced during the study period in AVNRT patients with ZF strategy (subgroup 1–6: 73 min vs. subgroup 7–12: 55.9, *p* = 0.008). In contrast, in AVNRT ablations with fluoroscopy procedural time remained constant (subgroup 1–6: 55.7 min vs. subgroup 7–12: 57.2 min, *p* = NS). At first, there were significant difference between AVNRT ZF subgroup 1–6: 73 min vs. F subgroup 1–6: 55.7 min, *p* = 0.03. By the end of the study period, this significant difference has become non-significant between ZF and F subgroups (ZF subgroups 7–12: 55.9 min vs. F subgroups 7–12: 57.2 min, *p* = NS) (Figure 4A).

In case of AFL, we did not observe the above pattern (Figure 4B). There is a decrease in procedure time over time in the AFL F subgroups (1–5: 54.4 min vs. 5–10: 44.4 min, *p* = 0.047).

Regarding the AP subgroup, ZF procedural times were reduced considerably during the second half of the study, as well (ZF subgroups 1–3: 86.2 min vs. ZF subgroups 4–7: 60.1, *p* = 0.077, Figure 4C). At the second half of the study, we observed that the procedure time was significantly lower in the ZF subgroup compared to F subgroup (ZF subgroup 4–7: 60.1 min vs. F subgroup 4–7: 85 min, *p* = 0.021).

As expected, in most cases where conversion to fluoroscopy was needed, procedural times were considerably longer through the whole study period in each arrhythmia subgroup.

## Discussion

Cardiac catheter ablation has emerged as a gold-standard therapeutic intervention in the field of electrophysiology for the management of various arrhythmias. Medical societies stress the importance of minimizing x-ray exposure during these procedures, taking interests of both patients and health care providers into account (4, 16). Efficacy and safety data of zero-fluoroscopy interventions suggest that this approach is feasible and safe (17–19). However, no guidelines are available on detailed regulation of these procedures (i.e., patient selection, type of procedure, material and personal settings, etc.). Furthermore, implementation of these new procedures requires new technical skills and effort resulting in considerable variability between operating centers and physicians.

In our study we analysed data from the first consecutive 382 patients undergoing ZF radiofrequency (RF) ablation of AVNRT, AFL or AP at our institute. As a control group within the same 51-months period, 223 patients with traditional fluoroscopic method were enrolled.

Importantly, acute procedural success rate was equally high with both approaches across all arrhythmia groups (above 97%) like in other studies (8, 17, 18, 20). Complication rates were not significantly different between the two groups and remained low (0.6%) across the total cohort, indicating that implication of the zero-fluoroscopy method is effective and safe.

In the absence of guidelines advising patient characteristics with favorable outcome for ZF procedures, operating physicians have to define the type of applied procedure. We attempted to characterize a patient population preferable for the ZF approach. The most important factor was the type of arrhythmia. While 72% of AVNRT patients and 75% of AP patients were ablated with ZF approach, only less than half (49%) of AFL patients went under ZF procedure. Not surprisingly, the AFL group exhibited a higher comorbidity burden compared to the AVNRT and AP groups, with a greater prevalence of chronic conditions such as chronic heart failure and ischemic heart disease. Furthermore, we found that procedures initiated during ongoing arrhythmia were more likely to start with fluoroscopy. The presence of cardiac implantable electronic devices also favored fluoroscopic approach for safety reasons. In AVNRT, predominantly younger patients were ablated with ZF methods.

Implementation of a new therapeutic approach is challenging. At the beginning of our observational period, out of 25 consecutive patients we applied zero-fluoroscopy only in 10 cases (40%). In contrast, 22 out of the last 31 enrolled patients were ablated with zero-fluoroscopy approach (71%). Our results are in line with a previous study describing a zero-fluoroscopic learning-curve (21). Upon separating the arrhythmia cohorts, especially AVNRT and AFL populations showed gradual increase in the use of the ZF technique. In both groups, by the end of the observational period the use of fluoroscopy was reduced by approximately 30%.

Previous studies investigating the ZF approach in various SVTs reported mean procedural durations ranging from around 50 to 129 min (10, 19, 22–24). Our findings align with this range, with our ZF cases ranging from 58 to 70 min on average. For AVNRT and AFL arrhythmias, procedures using the F approach were shorter than ZF cases. This may be attributed to the fact that the 3D EAM system's implementation coincided with the commencement of this study, requiring additional time for adaptation to the new technology in the above new indications, as well. However, based on our data, it takes only an average of 5.1 min longer to perform ZF procedures. We can effectively eliminate harmful ionizing radiation with procedures that are either similar or only marginally longer than fluoroscopy-guided procedures.

In AVNRT cases, ZF procedural times were significantly reduced during the second part of the study period by 7 min. In the second half of the patient cohort, procedural time became even 1 min longer with the fluoroscopy method on average. This observation emphasizes that in this arrhythmia group the zero-fluoroscopy method can be efficiently adapted by time.

Concerning the AP group, shorter average ZF procedure durations were detected compared to F. Upon analysis of patient subgroups in chronological order, the above observation was explained by the ZF procedural time which was greatly and significantly reduced (by 16 min) in the second half of the study. In these later patients, ZF-guided interventions became shorter, emphasizing the importance of training and acquired experience of the operating physicians.

Interestingly, regarding atrial flutter we recorded 8 min longer procedure times with ZF approach without improvement during the second half of the study period which may be attributed to the highly variable anatomy of the cavotricuspid isthmus (25, 26).

There were several cases where the operator initiated a procedure with ZF approach but decided subsequently to incorporate fluoroscopy during the intervention. Conversion rate was relatively low (AFL = 7%, AVNRT = 4%), being the highest—and remaining constant during the study period—in the AP group (27%). Since in many of the AP cases left-sided AP-induced arrhythmias were detected, in the absence of ICE, we punctured the transseptal wall under fluoroscopic guidance. Other studies also showed similar, varying rates of conversion (27–29).

It is important to note that procedure time was the longest in the conversion cases. This extended duration may be attributable to the conversion process itself: operators were not always equipped with lead aprons initially and were often tempted to resolve issues for a longer period before deciding to convert to fluoroscopy. Interestingly, in these converted ZF procedures, fluoroscopy time was even significantly shorter compared to traditional F procedures in the AVNRT and AP groups. This suggests that even if fluoroscopy is eventually required to complete the procedure, it is favorable to apply first the ZF approach. Furthermore, by increasing the frequency of ZF cases, proportion of procedures requiring conversion in the AVNRT and AFL group decreased during the second half of the observation period, indicating a successful learning period. In summary, it is advisable and feasible to implement the ZF approach over the fluoroscopy technique during AVNRT, AP and typical AFL ablation procedures; initially slightly longer procedures are predicted to become significantly shorter by time and experience (30).

Concerning the long-term follow-up (mean: 3.2 years), we observed 97% success rate for the total cohort at 3-months, 94% at 12-months and 93% at last medical follow-up. The AVNRT group displayed the most favorable long-term ablation outcomes with a 2% arrhythmia recurrence rate, aligning with international data (28, 31). Interestingly, the AP group demonstrated the highest recurrence rate (10% at 3-months and 12% at 12-months); while the small sample size of AP ablations might amplify the calculated recurrence percentages. Importantly, in these groups, procedures were similarly successful with or without fluoroscopy in long-term, as well (30).

Atrial flutter is associated with increased risk of cardiovascular events and mortality. Although recent guidelines recommend CTI RF ablation in symptomatic AFL patients for rhythm control, its long-term effects on mortality and clinical outcome are relatively less established (32). Recent data of 1,892 AFL patients suggests that cardiovascular (CV) mortality, CV death, heart failure requiring hospitalization and stroke are all significantly reduced after CTI ablation (32, 33). In our study, within AFL patients, arrhythmia recurrence occurred later, at 1 year with 7% and at 3 years with 10% rate in average. These data are similar to the result of a meta-analysis encompassing 155 studies with 9,942 patients where the 13-month recurrence rate was 10.9% (Table 3) (15). Interestingly, in our population original arrhythmia recurrence was significantly more frequent in the zero-fluoroscopy subgroup, although this difference seems to be present mostly after 3 years follow-up. An important clinical difference was the higher rate of diabetes mellitus and higher BMI in the ZF group, which might be a contributing factor to a less effective RF ablation of AFL due to a presumed thicker cavotricuspid isthmus in that group. Furthermore, the ablation technique is different while ablating a focal pathology as in AVNRT and AP. It cannot be excluded, that the operator had slightly different length of the RF ablation deliveries during linear ablation of the CTI depending on the mapping technique. This late presentation of arrhythmia recurrence might be related to the initial learning curve when ZF strategy has been introduced. The efficacy of the RF ablation could be possibly further enhanced by using contact-force catheters combined with different lesion indices such as ablation index or lesion size index (34, 35). Nevertheless, this observation is intriguing and should be confirmed on a larger cohort in the future. It is important to note, that despite frequent arrhythmia recurrence, survival data were similar between ablations with ZF or F guided approach. The AFL population was older and the burden of CV risk factors, co-morbidities and the incidence of structural heart disease and heart failure were increased. The above characteristics explain the highest detected mortality rate in our study: the 3 years mortality rate of AFL patients was 13%. The cause of death often had a non-cardiovascular origin, suggesting that this is an extremely fragile patient subgroup.

In our study, incidence of clinically detected new-onset atrial fibrillation was 7% in the total population during follow-up. The majority (86%) of noAF was detected in the AFL group; within these patients new arrhythmia development reached 15% (2.5%/year). Previously other studies have reported from 33% to 37% new-onset atrial fibrillation after CTI ablation (36). It is important to note, that in our patient population only clinically manifested AF episodes were registered, explaining lower incidence rates. New-onset atrial fibrillation rates were similar in ablations performed with either ZF or F approach (5% vs. 9%, respectively). Concerning traditional risk factors for AF, only previously detected pulmonary disorders were represented with higher frequency.

During follow-up, 5% of the total population required consequent cardiac device implantation. Importantly, there was no significant difference in pacemaker implantation rates between ZF and F cases (*n* = 18 vs. *n* = 15). Procedures were predominantly performed in AFL patients with an average 2%/year rate, indicating the above detailed frailty of this group. Indications were HFrEF diagnosis requiring primary or secondary prevention ICD or CRT devices, sick sinus syndrome and tachy-brady syndrome. The average time from ablation to pacemaker implantation was approximately one year (347 days), suggesting that PM implantations were not related to the index arrhythmia ablation.

Nevertheless, while our results focus on procedural outcomes such as acute success, complication rate and arrhythmia recurrence, we acknowledge that zero-fluoroscopy techniques may have broader benefits beyond procedural safety. Prior studies have suggested that ZF ablation may improve patient quality of life, reduce the need for repeat interventions, and offer economic advantages through decreased radiation infrastructure use and shorter post-procedural recovery (8). However, due to the retrospective nature of our study, these dimensions could not be assessed in detail. Further prospective research is needed to investigate these patient-centered and health-economic aspects more comprehensively.

In conclusion, this study contributes several novel insights to the current body of literature. We compared safety and short/long-term efficacy data of RF ablations guided by zero-fluoroscopy or fluoroscopy in 605 consecutive patients with AVNRT, AP, and AFL. Acute success rate, short-term complications and mortality rates were similar between patient groups in all SVTs, indicating that zero-fluoroscopy is a safe, efficient and feasible approach. Within our average 3.2-years follow-up, our study provides one of the longest follow-up datasets in real-world ZF ablation, enabling a meaningful comparison of long-term outcomes across different SVT types.

It highlights a potentially increased recurrence of atrial flutter following ZF ablation—an observation that warrants further investigation. Our structured analysis of the learning curve demonstrates how procedural efficiency and success evolve over time with zero-fluoroscopy adoption. The increasing frequency of ZF-guided procedures throughout the study period illustrates effective and practical implementation of this technique, offering valuable insights for other centers aiming to integrate ZF strategies into routine clinical practice.

Finally, mortality rate and long-term recurrence rate were highest in patients with AFL, meriting further investigation of this patient population. Mortality-, complication rates and occurrence of new-onset atrial fibrillation were identical in ZF vs. F guided procedures.

However—since recent data on mortality, arrhythmia recurrence and thromboembolic complication rates are missing in patients with RF ablation due to typical AFL—detailed analysis of applied oral anticoagulant therapy in respect to CHA_2_DS_2_-VASc and HAS-BLED scores should be performed.

## Limitations

Even though we enrolled a large patient cohort, our study is retrospective in nature. Medical follow-up data were partially obtained via the national electronic health care system and 30% of our patients were followed at our outpatient clinic. The use of ZF technique was completely based on the decision of the operator. While the choice of procedural strategy was at the discretion of the operator, and some differences in baseline characteristics were noted between groups, these did not appear to introduce systematic bias or significantly affect the outcome measures. Nevertheless, consecutive patient enrollment allowed us to document the increasing adoption of the ZF method in clinical routine. Due to technical conditions, we were not able to document the applied total radiation dose during fluoroscopy guided interventions. Furthermore, although the potential advantages of zero-fluoroscopy techniques regarding patient-reported outcomes and economic burden have been demonstrated in prior studies (e.g., Casella et al., NO-PARTY trial), these data were not available retrospectively in our setting. A prospective extension of our study incorporating standardized PRO questionnaires is currently in preparation, with institutional ethical approval underway. Finally, we recorded low frequency of ischaemic stroke after ablation of atrial flutter. Majority of our patients—despite the successful intervention—received extended oral anticoagulant therapy, most likely explaining low thromboembolic complication rates.

## Data Availability

The original contributions presented in the study are included in the article/Supplementary Material, further inquiries can be directed to the corresponding author.

## References

[1] Brugada J Katritsis DG Arbelo E Arribas F Bax JJ Blomström-Lundqvist C 2019 ESC guidelines for the management of patients with supraventricular tachycardiaThe task force for the management of patients with supraventricular tachycardia of the European society of cardiology (ESC). Eur Heart J. (2020) 41(5):655–720. 10.1093/eurheartj/ehz467 31504425

[2] Page RL Joglar JA Caldwell MA Calkins H Conti JB Deal BJ 2015 ACC/AHA/HRS guideline for the management of adult patients with supraventricular tachycardia: a report of the American college of cardiology/American heart association task force on clinical practice guidelines and the heart rhythm society. Circulation. (2016) 133(14):e506–74. 10.1161/CIR.0000000000000311 26399663

[3] Hirshfeld JW Jr. Balter S Brinker JA Kern MJ Klein LW Lindsay BD ACCF/AHA/HRS/SCAI clinical competence statement on physician knowledge to optimize patient safety and image quality in fluoroscopically guided invasive cardiovascular procedures: a report of the American college of cardiology foundation/American heart association/American college of physicians task force on clinical competence and training. Circulation. (2005) 111(4):511–32. 10.1161/01.CIR.0000157946.29224.5D 15687141

[4] Picano E Vañó E Rehani MM Cuocolo A Mont L Bodi V The appropriate and justified use of medical radiation in cardiovascular imaging: a position document of the ESC associations of cardiovascular imaging, percutaneous cardiovascular interventions and electrophysiology. Eur Heart J. (2014) 35(10):665–72. 10.1093/eurheartj/eht394 24401558

[5] Limacher MC Douglas PS Germano G Laskey WK Lindsay BD McKetty MH ACC Expert consensus document. Radiation safety in the practice of cardiology. J Am Coll Cardiol. (1998) 31(4):892–913. 10.1016/s0735-1097(98)00047-3 9525565

[6] Birnie D Healey JS Krahn AD Ahmad K Crystal E Khaykin Y Prevalence and risk factors for cervical and lumbar spondylosis in interventional electrophysiologists. J Cardiovasc Electrophysiol. (2011) 22(9):957–60. 10.1111/j.1540-8167.2011.02041.x 21385267

[7] LaPage MJ Saul JP. Update on rhythm mapping and catheter navigation. Curr Opin Cardiol. (2011) 26(2):79–85. 10.1097/HCO.0b013e3283437d48 21245755

[8] Casella M Dello Russo A Pelargonio G Del Greco M Zingarini G Piacenti M Near zerO fluoroscopic exPosure during catheter ablAtion of supRavenTricular arrhYthmias: the NO-PARTY multicentre randomized trial. Europace. (2016) 18(10):1565–72. 10.1093/europace/euv344 26559916 PMC5072134

[9] Earley MJ Showkathali R Alzetani M Kistler PM Gupta D Abrams DJ Radiofrequency ablation of arrhythmias guided by non-fluoroscopic catheter location: a prospective randomized trial. Eur Heart J. (2006) 27(10):1223–9. 10.1093/eurheartj/ehi834 16613932

[10] Seizer P Bucher V Frische C Heinzmann D Gramlich M Müller I Efficacy and safety of zero-fluoroscopy ablation for supraventricular tachycardias. Use of optional contact force measurement for zero-fluoroscopy ablation in a clinical routine setting. Herz. (2016) 41(3):241–5. 10.1007/s00059-015-4358-4 26462477

[11] Giaccardi M Del Rosso A Guarnaccia V Ballo P Mascia G Chiodi L Near-zero x-ray in arrhythmia ablation using a 3-dimensional electroanatomic mapping system: a multicenter experience. Heart Rhythm. (2016) 13(1):150–6. 10.1016/j.hrthm.2015.09.003 26341606

[12] Porter MJ Morton JB Denman R Lin AC Tierney S Santucci PA Influence of age and gender on the mechanism of supraventricular tachycardia. Heart Rhythm. (2004) 1(4):393–6. 10.1016/j.hrthm.2004.05.007 15851189

[13] Gist K Tigges C Smith G Clark J. Learning curve for zero-fluoroscopy catheter ablation of AVNRT: early versus late experience. Pacing Clin Electrophysiol. (2011) 34(3):264–8. 10.1111/j.1540-8159.2010.02952.x 21070259

[14] Chinitz JS Gerstenfeld EP Marchlinski FE Callans DJ. Atrial fibrillation is common after ablation of isolated atrial flutter during long-term follow-up. Heart Rhythm. (2007) 4(8):1029–33. 10.1016/j.hrthm.2007.04.002 17675077

[15] Pérez FJ Schubert CM Parvez B Pathak V Ellenbogen KA Wood MA. Long-term outcomes after catheter ablation of cavo-tricuspid isthmus dependent atrial flutter: a meta-analysis. Circ Arrhythm Electrophysiol. (2009) 2(4):393–401. 10.1161/CIRCEP.109.871665 19808495

[16] Faroux L Daval C Lesaffre F Blanpain T Chabert JP Martin A Physicians’ exposure to radiation during electrophysiology procedures. J Interv Card Electrophysiol. (2019) 55(2):233–7. 10.1007/s10840-019-00568-1 31177353

[17] Debreceni D Janosi K Vamos M Komocsi A Simor T Kupo P. Zero and minimal fluoroscopic approaches during ablation of supraventricular tachycardias: a systematic review and meta-analysis. Front Cardiovasc Med. (2022) 9:856145. 10.3389/fcvm.2022.856145 35479287 PMC9037593

[18] Fadhle A Hu M Wang Y. The safety and efficacy of zero-fluoroscopy ablation versus conventional ablation in patients with supraventricular tachycardia. Kardiol Pol. (2020) 78(6):552–8. 10.33963/KP.15293 32301592

[19] Giaccardi M Mascia G Paoletti Perini A Giomi A Cartei S Milli M. Long-term outcomes after “Zero X-ray” arrhythmia ablation. J Interv Card Electrophysiol. (2019) 54(1):43–8. 10.1007/s10840-018-0390-7 29948584

[20] Bergonti M Dello Russo A Sicuso R Ribatti V Compagnucci P Catto V Long-term outcomes of near-zero radiation ablation of paroxysmal supraventricular tachycardia: a comparison with fluoroscopy-guided approach. JACC Clin Electrophysiol. (2021) 7(9):1108–17. 10.1016/j.jacep.2021.02.017 33933407

[21] Santoro A Di Clemente F Baiocchi C Zacà V Bianchi C Bellini C From near-zero to zero fluoroscopy catheter ablation procedures. J Cardiovasc Electrophysiol. (2019) 30(11):2397–404. 10.1111/jce.14121 31424119

[22] Tseng WC Wu MH Lu CW Wu KL Wang JK Lin MT Zero fluoroscopy during ablation of right-sided supraventricular tachycardia substrates in a pediatric population—initial experience in Taiwan. Acta Cardiol Sin. (2019) 35(5):476–83. 10.6515/ACS.201909_35(5).20190211A 31571796 PMC6760136

[23] Chen G Wang Y Proietti R Wang X Ouyang F Ma CS Zero-fluoroscopy approach for ablation of supraventricular tachycardia using the ensite NavX system: a multicenter experience. BMC Cardiovasc Disord. (2020) 20(1):48. 10.1186/s12872-020-01344-0 32013865 PMC6996189

[24] Troisi F Guida P Quadrini F Di Monaco A Vitulano N Caruso R Zero fluoroscopy arrhythmias catheter ablation: a trend toward more frequent practice in a high-volume center. Front Cardiovasc Med. (2022) 9:804424. 10.3389/fcvm.2022.804424 35571172 PMC9095839

[25] Baccillieri MS Rizzo S De Gaspari M Paradiso B Thiene G Verlato R Anatomy of the cavotricuspid isthmus for radiofrequency ablation in typical atrial flutter. Heart Rhythm. (2019) 16(11):1611–8. 10.1016/j.hrthm.2019.05.030 31150815

[26] Regoli F Faletra F Marcon S Leo LA Dequarti MC Caputo ML Anatomic characterization of cavotricuspid isthmus by 3D transesophageal echocardiography in patients undergoing radiofrequency ablation of typical atrial flutter. Eur Heart J Cardiovasc Imaging. (2018) 19(1):84–91. 10.1093/ehjci/jew336 28180237

[27] Morka A Sledz J Deutsch K Ludwik B Zagrodzka M Szydlowski L Feasibility and performance of catheter ablation with zero-fluoroscopy approach for regular supraventricular tachycardia in patients with structural and/or congenital heart disease. Medicine. (2019) 98(41):e17333. 10.1097/MD.0000000000017333 31593082 PMC6799864

[28] Świętoniowska-Mścisz A Stec P Stec S Szydłowski L Zagrodzka M Kusa J Efficacy and safety of zero-fluoroscopy approach for ablation of atrioventricular nodal reentry tachycardia: experience from more than 1000 cases. J Interv Card Electrophysiol. (2023) 66(5):1231–42. 10.1007/s10840-022-01419-2 36495412

[29] Walsh KA Galvin J Keaney J Keelan E Szeplaki G. First experience with zero-fluoroscopic ablation for supraventricular tachycardias using a novel impedance and magnetic-field-based mapping system. Clin Res Cardiol. (2018) 107(7):578–85. 10.1007/s00392-018-1220-8 29476203 PMC6002461

[30] Prolič Kalinšek T Šorli J Jan M Šinkovec M Antolič B Klemen L Conventional fluoroscopy-guided versus zero-fluoroscopy catheter ablation of supraventricular tachycardias. BMC Cardiovasc Disord. (2022) 22(1):98. 10.1186/s12872-022-02544-6 35282836 PMC8919640

[31] Razminia M Willoughby MC Demo H Keshmiri H Wang T D’Silva OJ Fluoroless catheter ablation of cardiac arrhythmias: a 5-year experience. Pacing Clin Electrophysiol. (2017) 40(4):425–33. 10.1111/pace.13038 28160298

[32] Yugo D Chen YY Lin YJ Chien KL Chang SL Lo LW Long-term mortality and cardiovascular outcomes in patients with atrial flutter after catheter ablation. Europace. (2022) 24(6):970–8. 10.1093/europace/euab308 34939091

[33] Tomson TT Kapa S Bala R Riley MP Lin D Epstein AE Risk of stroke and atrial fibrillation after radiofrequency catheter ablation of typical atrial flutter. Heart Rhythm. (2012) 9(11):1779–84. 10.1016/j.hrthm.2012.07.013 22813577

[34] Compagnucci P Dello Russo A Bergonti M Anselmino M Zucchelli G Gasperetti A Ablation index predicts successful ablation of focal atrial tachycardia: results of a multicenter study. J Clin Med. (2022) 11(7):1802. 10.3390/jcm11071802 35407408 PMC8999753

[35] Boles U Gul EE Fitzpatrick N Enriquez A Conroy J Ghassemian A Lesion size index in maximum voltage-guided cavotricuspid ablation for atrial flutter. J Innov Card Rhythm Manag. (2017) 8(6):2732–8. 10.19102/icrm.2017.080603 32494452 PMC7252914

[36] Seara JG Roubin SR Gude Sampedro F Barreiro VB Sande JM Mañero MR Risk of atrial fibrillation, stroke, and death after radiofrequency catheter ablation of typical atrial flutter. Clin Res Cardiol. (2014) 103(7):543–52. 10.1007/s00392-014-0682-6 24566731

